# The C.O.S. as Collector for Charities

**Published:** 1891-01-03

**Authors:** 


### Passing Topics.

### THE C.O.S. AS COLLECTOR FOR CHARITIES.

Now and again a reference has appeared, more or less prominently, to a proposal of the Charity Organization Society, to undertake the part of middlemen between the charities and the subscribers who support them, and we have before us a copy of a circular letter dated December 20th, wherein the Secretary of the Society is "desired to draw your (the subscriber's) attention to an arrangement which has been in operation for some years by which any number of subscriptions to Metropolitan Charities can be effected by a single payment through this Society, who would act as a Clearing House." It is obligingly added that "if after reading the enclosed paper you would wish to avail yourself of this branch of the Society's work. . . I shall be glad if you will communicate with me."

We think it a pity that this proposal should not receive all the publicity it merits, and while placing it before our readers, we may perhaps express a hope that some subscribers who are not eagerly anxious to avail themselves of the offer will also communicate with the secretary, and, in courteous terms, of course, desire he will mind his own business. We are bold enough to think that few more unhappy suggestions have been made than that the offerings to our Institutions instinct with the individuality of the contributors, and bright from sympathy's mint, should rest for a moment in the chill palm of the C.O.S.

If we could believe it possible that the future of our great philanthropic undertakings is likely to be made dependent upon the good offices of the society, the outlook would indeed be serious. But, happily, there is enough of life and warmth in the stream of Charity to protect it from the frigid influences of the Arctic Organisation of Buckingham Street. The overwhelming majority of the men and women who support our institutions are little likely to welcome a suggestion designed to relieve them of the precious privilege of helping onward with their own hands and out of the fulness of their own warm hearts the great philanthropic works with which they have identified themselves.

It is one of the misfortunes even of the best institutional system that too much must be done by deputy. The cry of all intelligent managers is, Give us more of your real earnest personal interest; come and see for yourselves what manner of work it is your money is helping to carry on. It has been often asserted—and the assertion is true enough to bear repetition—that the most prosperous of the charitable institutions of the Victorian era are those which have secured most largely the personal concern and services of their founders and promotors, and whose managers cordially welcome and assiduously cultivate the solicitude of their supporters. When the secretary, in his desire to exercise patronage, invites subscribers to constitute his society their almoner and intermediary, he altogether overlooks the important fact, patent to everybody else, that even could he succeed he would soon be confronted by the additional and more stupendous task of how to maintain the subscriptions while annihilating the conditions needful to their production. The graces of charity are born of a genial temperament, and when subjected to adverse influences soon disappear. It has never been contended even by its staunchest friends that the C. O. S. is calculated to stimulate philanthropy into activity. The publication of this circular fairly entitles us to ask whether the suggestion it contains is not one which strikes—though we hope harmlessly—at the root of support "by voluntary contribution?" Agricola.

